# Integration of microarray data and literature mining identifies a sex bias in DPP4+CD4+ T cells in HIV-1 infection

**DOI:** 10.1371/journal.pone.0239399

**Published:** 2020-09-18

**Authors:** Hans Christian Stubbe, Christine Dahlke, Katharina Rotheneder, Renate Stirner, Julia Roider, Raffaele Conca, Ulrich Seybold, Johannes Bogner, Marylyn Martina Addo, Rika Draenert

**Affiliations:** 1 Division of Infectious Diseases, Department of Medicine IV, Hospital of the LMU Munich, Munich, Germany; 2 German Center for Infection Research (DZIF), Partner Site Munich, Germany; 3 Department of Medicine II, Hospital of the LMU Munich, Munich, Germany; 4 Division of Infectious Diseases, First Department of Medicine, University Medical Center Hamburg-Eppendorf, Hamburg, Germany; 5 German Center for Infection Research (DZIF), Partner Site Hamburg-Lübeck-Borstel-Riems, Germany; 6 Department of Clinical Immunology of Infectious Diseases, Bernhard Nocht Institute for Tropical Medicine, Hamburg, Germany; 7 Department of Pediatrics Dr. Von Hauner Children's Hospital, Hospital of the LMU Munich, Munich, Germany; 8 Antibiotic Stewardship Team, Hospital of the LMU Munich, Munich, Germany; University of Pittsburgh, UNITED STATES

## Abstract

HIV-1 infection exhibits a significant sex bias. This study aimed at identifying and examining lymphocyte associated sex differences in HIV-1 pathogenesis using a data-driven approach. To select targets for investigating sex differences in lymphocytes, data of microarray experiments and literature mining were integrated. Data from three large-scale microarray experiments were obtained from NCBI/GEO and screened for sex differences in gene expression. Literature mining was employed to identify sex biased genes in the microarray data, which were relevant to HIV-1 pathogenesis and lymphocyte biology. Sex differences in gene expression of selected genes were investigated by RT-qPCR and flowcytometry in healthy individuals and persons living with HIV-1. A significant and consistent sex bias was identified in 31 genes, the majority of which were related to immunity and expressed at higher levels in women. Using literature mining, three genes (DPP4, FCGR1A and SOCS3) were selected for analysis by qPCR because of their relevance to HIV, as well as, B and T cell biology. DPP4 exhibited the most significant sex bias in mRNA expression (p = 0.00029). Therefore, its expression was further analyzed on B and T cells using flowcytometry. In HIV-1 infected controllers and healthy individuals, frequencies of CD4+DPP4+ T cells were higher in women compared to men (p = 0.037 and p = 0.027). In women, CD4 T cell counts correlated with a predominant decreased in DPP4+CD4+ T cells (p = 0.0032). Sex differences in DPP4 expression abrogated in progressive HIV-1 infection. In conclusion, we found sex differences in the pathobiology of T cells in HIV-1 infection using a data-driven approach. Our results indicate that DPP4 expression on CD4+ T cells might contribute to the immunological sex differences observed in chronic HIV‑1 infection.

## Introduction

Numerous studies provide evidence that the human immunodeficiency virus 1 (HIV-1) infection is sex biased. In women, lower viral loads and higher CD4 T cell counts are measured in all stages of the disease. At the same viral load, women are at a 1.6-fold higher risk of progression to AIDS compared to men [1]. Various factors contribute to these differences. A major role is attributed to sex differences in the immunity against HIV-1 [2].

HIV-1 infection profoundly interferes with the immune system and elicits persistent immune activation and inflammation, which are associated with progression to AIDS. A critical driver of this pathology is the interferon response of plasmacytoid dendritic cells (pDC) to HIV-1: sensing of viral components elicits an interferon alpha response by pDC, which is stronger in women compared to men [3]. This translates to an increased immune activation and inflammation in women with downstream effects on components of the adaptive immune system such as B or T cells [3, 4]. B cells and more importantly T cells play a pivotal role in the pathogenesis of HIV-1 infection [5]. Also, they are subject to immunological sex differences [6]. A better understanding of sex differences in the pathogenesis of HIV-1 infection warrants the investigation of sex differences in B and T cells in HIV-1 infection. Therefore, this study aimed at identifying sex differences in B and T cell biology and investigating their relationship to markers of disease progression.

**Fig 1:**
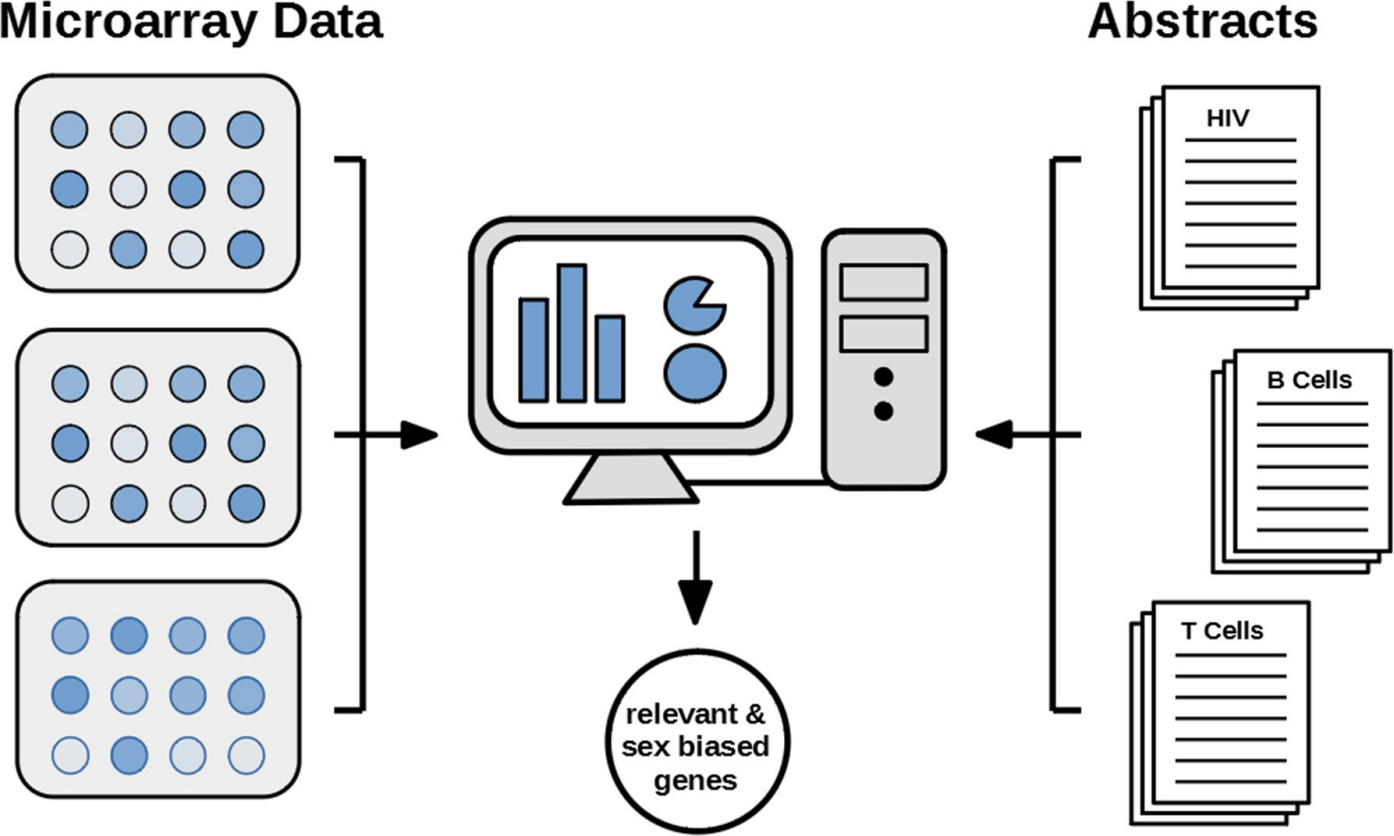
Identification of sex biased genes using gene expression data and literature mining. Screening of microarray gene expression profiles for sex biased genes and literature mining of abstracts obtained from PubMed identified genes which were sex biased and relevant for HIV and B or T cell biology.

Gene expression profiles obtained from the NCBI Genome Expression Omnibus (NCBI/GEO) and literature mining were integrated to identify candidate genes for analysis by reverse transcription quantitative polymerase chain reaction (RT-qPCR) and flowcytometry (Fig 1). We identified a sex bias in *dipeptidyl peptidase 4* (*DPP4*) expression in T cells, which correlated with markers of disease progression. These data indicate a new line of research on the sex bias in HIV-1 pathogenesis.

## Materials and methods

### Screening of microarray data for sex differences

B and T cells constitute a significant quantity of cells circulating in the peripheral blood. We assumed that a sex bias in their gene expression might be reflected in an overall sex bias in the peripheral blood. Therefore, three large-scale and publicly available gene expression profiling microarray datasets of the peripheral blood were investigated for sex differences in gene expression.

Datasets were selected based on the following criteria: The datasets had to be derived from single-color gene expression profiling by microarray. Experimental design had to be available. Information on the corresponding data analysis pipeline had to be accessible and the data had to be preprocessed, normalized and quality checked. The samples had to be acquired by PaxGene tubes from whole blood. Information on sex, age and health status had to be available for each sample. The included samples had to be obtained from healthy individuals aged between 17 and 43 years of age. The datasets had to include at least ten samples of each, female and male donors. Three single-color microarray gene-profiling datasets matching these criteria were selected and downloaded from NCBI/GEO [7].

The respective datasets were provided to NCBI/GEO by Joseph E. Powell from Brisbane (Australia, GSE53195) [8–10], Thomas L. Ortel from Durham (USA, GSE19151) [11] and Guillemette Duchateau-Nguyen from Basel (Switzerland, GSE16028) [12]. In total, 245 gene expression profiles of healthy individuals (HI) aged between 17 and 43 years were analyzed for sex differences in gene expression employing the GEO2R algorithm (NCBI/GEO). Of these samples, 53% were obtained from women.

The respective analyses were carried out using R/Bioconductor software [13]. The GEO2R script was used to investigate the microarray datasets obtained from NCBI/GEO [14, 15]. Sex biased genes were filtered for consistent up or downregulation in the three microarray datasets and for statistically significant differences in gene expression. To reduce the number of false positives, only genes were selected, which were highly significantly sex biased (p ≤ 0.01) in at least two of the three datasets and significantly sex biased (p ≤ 0.05) in all datasets.

### Analysis of gene ontology categories

Gene ontology (GO) category analysis was used to investigate the biological context of sex biased genes observed in the microarray data. GO analysis was performed using the Molecular Signatures Database published and maintained by the Broad Institute [16].

### Literature mining of sex biased genes

An algorithm written in R was used to count occurrences of sex biased genes in distinct sets of abstracts about HIV, B cells and T cells (S1 Fig, R code in S1 File). The abstracts were obtained from PubMed. To identify gene occurrences, each set of abstracts was searched for the occurrence of gene names, symbols, and aliases of the respective genes. The required symbols, names and aliases of genes were obtained from the Human Genome Organization’s (HUGO) Gene Nomenclature Committee (HGNC) webpage [17]. Test datasets were used to optimize the algorithm’s performance and to test its specificity and sensitivity. A specificity of 99.71% could be achieved at a sensitivity of 97.34%. PubMed was searched for publications about HIV, B and T cells (S2 Table). The respective abstracts were searched for occurrences of sex biased genes as observed in the microarray. Genes occurring in abstracts about HIV and B or T cells were selected for subsequent analysis by RT-qPCR and flowcytometry.

### Patient samples for RT-qPCR and flowcytometry

Altogether, 33 healthy individuals (HI) and 68 persons living with HIV-1 (PLHIV) were included at the University Hospitals in Munich and Hamburg (Table 1). All were included after informed consent was obtained. All participants were of full age (≥ 18 years) and the groups were age matched. PLHIV were treatment-naïve and started combined antiretroviral therapy (cART) shortly after blood draw. PLHIV comprised two subgroups: 24 controllers with stable disease (stable CD4 T cell counts, stable HIV viral loads and no occurrence of HIV associated or AIDS defining diseases in follow-up visits), CD4 T cell counts greater than 500 cells/μl and average viral loads lower than 5,000 copies/ml (some exhibited slightly higher viral load at blood draw but not at earlier and later visits) in the absence of antiretroviral treatment and 44 progressors not meeting these criteria. Blood was collected by venipuncture. Protocols were approved by the ethics committees of the University of Hamburg and the LMU Munich.

**Table 1:** Samples for RT-qPCR and flowcytometry.

	*Flowcytometry*						*RT-qPCR*	
*Cohort*	Munich						Hamburg	
*Group*	Healthy		Controller		Progressor		Healthy	
*Sex*	Female	Male	Female	Male	Female	Male	Female	Male
*Number [n]*	10	9	14	10	19	25	7	7
*VL [copies/ml] ^¶^*	-	-	2079	2206	23298	37057	-	-
			[1014, 3562]	[1065, 8554]	[13246, 54602]	[19819, 97109]		
*CD4 [cells/μl] ^¶^*	-	-	574	631	327	305	-	-
			[503, 711]	[549, 656]	[156, 455]	[260, 417]		
*Age [years]^¶^*	29	30	34	40	37	38	29	26
	[24, 49]	[28, 32]	[30, 44]	[27, 42]	[29, 43]	[32, 48]	[28, 31]	[24, 27]
*Ethnicity/Race*								
*African*	0	0	1	0	0	0	0	0
*Caucasian*	10	9	13	10	19	25	7	7

The table shows the HI and PLWH who were included in this analysis. Samples for RT-qPCR were obtained in Hamburg, while all other samples were obtained in Munich.

^¶^Values as medians with inter-quartile ranges.

### Analysis of gene expression by RT-qPCR

RT-qPCR was carried out using freshly isolated peripheral blood mononuclear cells (PBMC) as described elsewhere [18]. In short, PBMC were obtained using Ficoll-density gradient centrifugation. RNA was extracted from 10^6 PBMCs using a Trizol based protocol. cDNA was synthesized using Superscipt III (Invitrogen) with random hexamer primers as per manufacturer’s instruction. The RT-qPCR was carried out using the SYBR Green Mastermix (ThermoFisher) and specific primers (S3 Table). Samples were measured on the Rotor-Gene Q (Qiagen). Relative mRNA expression was computed with the R/Bioconductor/EasyqpcR software using the qBase algorithm and normalizing on the housekeeping genes *HPRT1* and *HMBS* [19].

### Surface expression of DPP4 on lymphocyte subsets

Cryopreserved PBMC were analyzed by flowcytometry on a LSRFortessa (BD) using the viability dye ZombieNIR and the fluorochrome-conjugated antibodies FITC-CD3, PECy7-CD4, PerCP-CD8, BV510-CD19, BV421-CD38 and PE-CD26/DPP4 from BioLegend. Data was analyzed with R/Bioconductor as outlined by O’Neill et al. (gating hierarchy and exemplary gates in S2 Fig) [20].

### Statistical analysis

Statistical analysis was performed using the R programming language (version 3.6). Differences in medians were tested using the Mann-Whitney U test. If the test hypothesis was one-sided, a one-sided test was chosen. Correlations were identified computing Spearman’s rho. P-values were adjusted for multiple comparisons using the Benjamini-Hochberg correction, if applicable and referred to as q-values.

## Results

### Sex differences in microarray data

**Fig 2:**
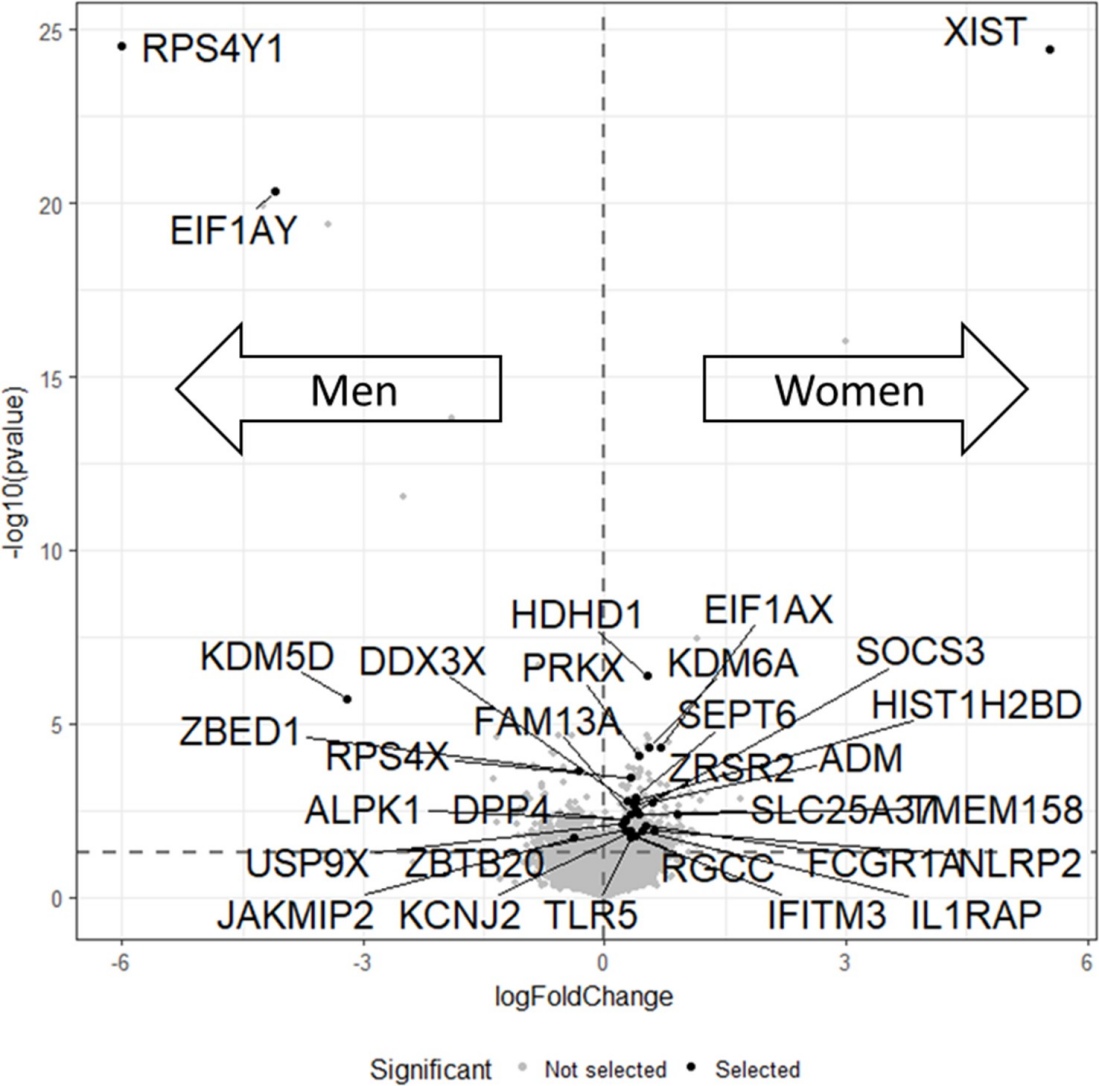
Sex differences in gene expression in three microarray experiments. Volcano plot with fold changes over p-values. Data of three independent microarray experiments were screened for sex differences. Means were computed for fold changes and p-values. Fold changes are displayed on a logarithmic scale and p-values on a scale of the negative decadic logarithms. Points right of the vertical dashed line correspond to higher expression in women and vice versa. The horizontal dashed line indicates the significance level 0.05 with points above this line representing an average p-value < 0.05. Genes, which were significantly and consistently differentially expressed in all three experiments are represented as black dots with names. They were selected for GO Category analysis and literature mining.

B and T cells account for a significant number of leukocytes [21]. A sex bias in their gene expression might be reflected in the overall gene expression in the peripheral blood. To identify sex biased genes, sex differences in gene expression were computed for three microarray datasets obtained from NCBI/GEO. Of 24,449 investigated genes, only genes were selected which were highly significantly sex biased (*p* ≤ 0.01) in at least two of the three datasets and significantly sex-biased (*p* ≤ 0.05) in all datasets. Some genes were inconsistently sex biased comparing the three datasets (i.e. higher expression in women in one dataset, higher expression in men, in the other) and were removed. Overall, this yielded 31 sex biased genes (Fig 2, S3 Fig).

GO category analysis was used to examine the general context of the 31 sex biased genes. The strongest overlaps of the 31 sex biased genes with GO categories were found for the categories “*GO_IMMUNE_RESPONSE”* (q = 0.0001), “*GO_IMMUNE_SYSTEM_PROCESS”* (q = 0.0001) and “*GO_DEFENSE_RESPONSE”* (q = 0.0002). These categories strongly related to the immune system and the immune response. Altogether, 11 of 31 genes were associated with any process related to the development of functioning of the immune system (S1 Table).

### Literature mining of sex biased genes

PubMed was searched for publications about HIV, B and T cells and the respective abstracts were downloaded. This produced three sets encompassing a total of 221,326 abstracts (S2 Table). The 31 sex biased genes were analyzed for occurrences in each set of abstracts. Six genes occurred at least once in abstracts about HIV and B or T cells (S4 Table). The three genes with the lowest average *p-*values comparing the three microarray datasets (*SOCS3*, *DPP4* and *FCGR1A*) were selected for analysis by RT-qPCR.

### Sex bias in the expression of *DPP4*, *FCGR1A* and *SOCS3* in healthy individuals

**Fig 3:**
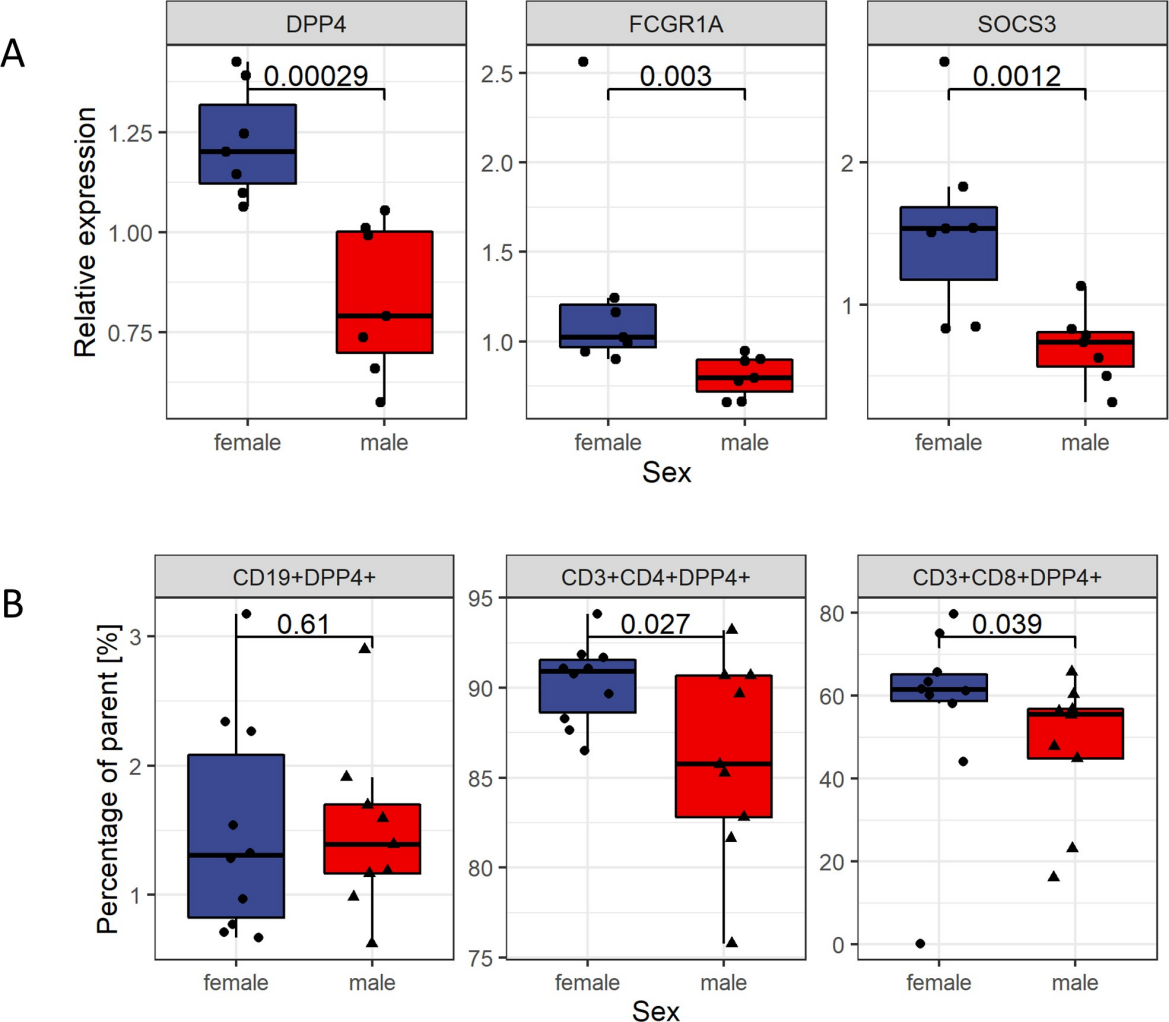
Sex biased genes in healthy individuals. (A) The three most significantly sex biased genes were selected for analysis by RT-qPCR. Sex differences were assessed in PBMC of 14 healthy individuals (7 women, 7 men). Relative mRNA expression was computed using the qBase algorithm normalizing on the housekeeping genes *HPRT1* and *HMBS*. A one-sided Wilcoxon rank sum test was used to test for higher gene-expression in women as observed in the microarray data. (B) DPP4 surface expression on T cells was measured by flowcytometry in PBMC obtained from 19 healthy donors (10 women, 9 men). Frequencies were calculated as percentage of the parent population. Percentages of DPP4 positive cells were compared between women and men. A one-sided Wilcoxon signed rank test was used to test for higher frequencies of DPP4+ T cells in women as hypothesized from microarray and RT-qPCR data.

Gene expression of *DPP4*, *FCGR1A* and *SOCS3* was analyzed in PBMC obtained from HI by RT-qPCR. In accordance with the microarray data, it was hypothesized that mRNA-expression was higher in women. Indeed, relative mRNA expression of *DPP4*, *FCGR1A* and *SOCS3* was higher in women compared to men (*p* = 0.00029, *p* = 0.003 and *p* = 0.001, respectively; Fig 3A).

### DPP4+ T cells in healthy individuals

The sex bias in gene expression measured by RT-qPCR was statistically most significant for *DPP4* (Fig 3A). DPP4, also known as CD26, occurs integrated into the cellular membrane of many tissues or as soluble form in virtually all body-fluids. On B and T cells, engagement of DPP4 promotes a co-stimulatory signal and its surface expression is associated with B and T cell activation [22]. Therefore, DPP4 expression was analyzed on B and T cells of HI by flowcytometry. Since a higher *DPP4*-mRNA expression was observed in women in both RT-qPCR and microarray data, we hypothesized, that DPP4 would be expressed at higher rates on B or T cells obtained from healthy women compared to men. In fact, higher frequencies of CD4+DPP4+ and CD8+DPP4+ T cells were observed in women (90.9% [88.6% - 91.5%] vs. 85.76% [82.8%, 90.7%]; *p* = 0.027 and 61.5% [58.7%, 65.2%] vs. 55.5% [44.9%, 56.8%]; *p* = 0.039). For B cells, no sex bias was found (Fig 3B).

### DPP4+ T cells in HIV-1 infection

**Fig 4:**
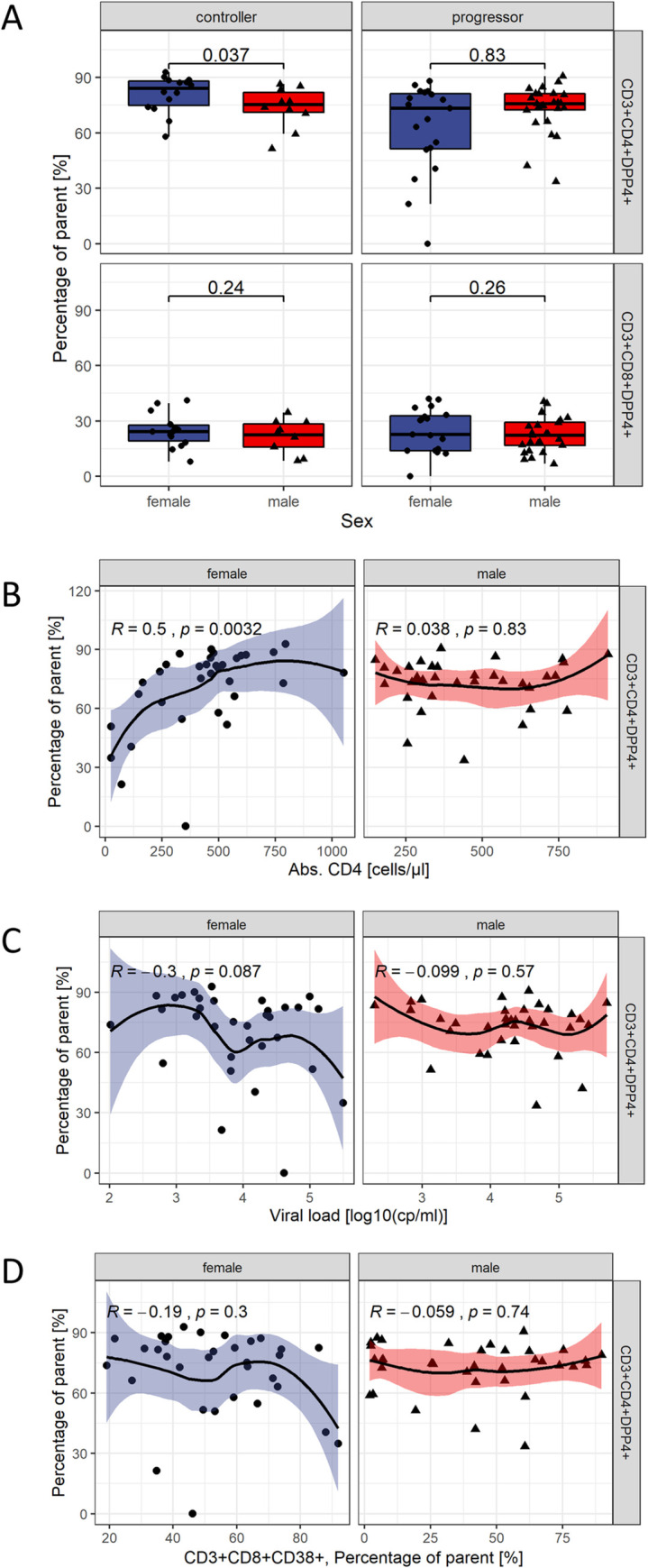
DPP4+ T cells in PLHIV. (A) Expression of DPP4 on CD4 T cells was measured in HIV-1 positive patients by flowcytometry in PBMC. PBMC were obtained from 19 healthy individuals (10 women, 9 men), 24 controllers (14 women, 10 men) and 44 progressors (19 women, 25 men). Percentages of DPP4 positive CD4 T cells were compared between female and male healthy controls, controllers and progressors. Differences were tested for statistical significance using a one-sided Wilcoxon signed rank test testing for higher frequencies in women. (B-D) DPP4+CD4+ T cells were correlated with absolute CD4 T cell counts, HIV plasma viral load and CD4 T cell activation as measured by CD38. Samples were obtained from 68 persons living with HIV-1 (33 women, 35 men). Loess regression lines with 95% confidence intervals were added. The correlation coefficient r and the *p-*value were computed using the Spearman method.

Overall, lower frequencies of CD4+DPP4+ T cells were observed in PLHIV compared to healthy controls. Female progressors exhibited lower CD4+DPP4+ frequencies than female controllers (74.4% [51.6%, 81.7%] vs. 84% [75%, 88.1%], *p* = 0.01; S4 Fig), while no such difference was found in male controllers. Accordingly, significantly higher frequencies of CD4+DPP4+ T cells were observed in female controllers compared to male controllers (84% [75%, 88.1%] and 75.3% [71.1%, 81.8%], respectively; *p* = 0.037; Fig 4A), while no sex bias was observed in progressors. The percentages of CD8+ T cells expressing DPP4 were much lower in PLHIV compared to HI. Overall, this was more pronounced than in CD4+DPP4+ T cells (S4 Fig). Even though higher frequencies of CD8+DPP4+ T cells were observed in female HI compared to male HI, no sex differences were seen in this population among PLHIV (Fig 4A).

### DPP4+ T cells and markers of disease progression

Since lower frequencies of CD4+DPP4+ T cells were observed in progressors, the relationship of CD4+DPP4+ T cells with markers of disease progression was assessed in PLHIV. Plasma viral load, absolute CD4 T cell counts and CD8+CD38+ T cells are associated with disease progression [5, 23]. Overall, frequencies of CD4+DPP4+ T cells correlated with absolute CD4 T cell counts (r = 0.3; *q* = 0.026), while no statistically significant correlations were observed with viral load or T cell activation (S5 Fig). When examining these relationships separately for both sexes, strong positive correlations were observed for CD4+DPP4+ T cells with CD4 T cell counts in women (Spearman’s r = 0.50, *p* = 0.0032), but not in men (r = 0.038, *p* = 0.83) (Fig 4B). This association remained significant after adjusting for multiple comparisons (*q* = 0.019). Conversely, a trend towards a negative correlation of viral load and DPP4+CD4+ T cells was observed in women (r = -0.3, *p* = 0.087) but not in men (r = -0.099, *p* = 0.57) (Fig 4C). CD4+DPP4+ T cell frequencies did not correlate with frequencies of CD8+CD38+ T cells (Fig 4D). Intriguingly, CD4+DPP4+ T cell frequencies correlated positively with frequencies of CD4+CD38+ T cells in women (r = 0.49, *p* = 0.0036), but not in men (r = 0.13, *p* = 0.46) (S6 Fig).

## Discussion

In 2018, about 38 million people lived with HIV, approximately half of whom were women [24]. To date, few studies examined sex differences in the immune response against HIV-1. T cells play a crucial role in HIV-1 pathogenesis [5]. Here, we observed a yet unknown sex bias in DPP4+CD4+ T cells.

As previously reported, a significant number of sex biased genes of the peripheral blood is associated with the immune system and expressed at higher levels in women. These genes include autosomal as well as X- and Y-linked genes. Accordingly, GO category analysis of the 31 sex biased genes, which were identified in this study, were immunity associated: 11 of the 31 genes were related to the development or functioning of the immune system. In addition, most of these genes were expressed at higher rates in women compared to men.

Integration of microarray data with literature mining identified six of the 31 sex biased genes, which were relevant in HIV pathogenesis, and linked to B and T cell biology. The three most significantly sex biased genes were investigated by RT-qPCR confirming the sex bias observed in the microarray data. While no sex bias had been previously reported for *FCGR1A* and *SOCS3*, Pérez-Durillo et al. observed higher plasma levels of the soluble protein dipeptidyl peptidase 4 (DPP4) in women compared to men [25]. In our data, *DPP4* exhibited the most significant sex bias in mRNA expression of the three genes as tested by RT-qPCR.

The gene *DPP4* is located on long arm of chromosome 2 [26]. It encodes the protein dipeptidyl peptidase 4 (DPP4), also known as CD26 or adenosine deaminase complexing protein 2 (ADCP2) [27]. DPP4 exhibits peptidase activity and occurs integrated into the cellular membrane of many tissues and cell types or as soluble form in virtually all body-fluids [28]. Multiple studies support the role of *DPP4* in immunity through peptidase activity and as a cell-surface receptor. Therefore, we investigated DPP4 cell surface expression on B and T cells by flowcytometry. We observed higher frequencies of DPP4 on T cells obtained from healthy women compared to men. On T cells, DPP4 expression is associated with T cell activation [22]. Generally, T cell activation is stronger in women [6]. Higher DPP4 expression on T cells of women might reflect stronger T cell activation in women [22].

Data on the role of DPP4 in HIV-1 infection is conflicting. DPP4 expression on T cells has been linked to susceptibility of T cells to viral entry [29, 30]. Cleavage of C-C motif chemokine 5 (CCL5) by DPP4 increases the anti-viral effect of CCL5, while cleavage of stromal cell-derived factor-1 (SDF-1) mitigates its antiviral activity [31]. The HIV proteins tat and gp120 inhibit DPP4 function. Previous clinical studies found that inhibition of DPP4 by specific DPP4 inhibitors resulted in slightly increased rates of infectious diseases and might impair T cell response and function, while T cell frequencies were not affected [22, 32]. Interestingly, high DPP4 expression might protect from contracting HIV infection. Female sex workers exposed to HIV, who were not infected with HIV, expressed higher levels of DPP4+ T cells compared to healthy female controls [33]. Together, these observations suggest that DPP4 promotes a robust anti-viral immune response.

In PLHIV, lower frequencies of DPP4+ T cells occur very early during HIV infection and are not reversed by cART [22, 34]. In accordance with these observations, we found lower frequencies of CD4+ DPP4+ and CD8+DPP4+ T cells in PLHIV compared to HI. DPP4 is highly expressed on Th17 T cells [35]. Possibly, low frequencies of DPP4 T cells in HIV-1 infection are be due to the early loss of Th17 T cells in HIV infection [36]. Another conceivable cause of reduced DPP4+ T cell frequencies might lie in the early infection and destruction of memory/helper T cells expressing the CD4+CD45RO+CD26+ phenotype [31]. However, both theories cannot fully explain the reduction DPP4+ T cells within the CD8+ subset.

Intriguingly, we found that CD4+DPP4+ T cells were sex biased in HI and controllers, but not in progressors. Previous findings showed that sex differences in plasma viral load were less pronounced in advanced HIV-1 infection [37]. Our data suggest that this might be reflected in the absence of sex bias in CD4+DPP4+ T cells among progressors contrasting the sex bias which can be observed among HI and healthy individuals. For CD8+DPP4+ T cells, no sex bias was observed among PLHIV, while HI exhibited higher frequencies in women compared to men. Possibly this is due to the strong reduction of CD8+DPP4+ T cell percentages in HIV-1 infection, which was more pronounced for CD8+ T cells than for CD4+ T cells. In consequence, the effect size of a sex bias in percentages of CD8+DPP4+ T cells might be smaller or absent and therefore might have escaped detection in our study.

Given the decrease of CD4+DPP4+ T cells in progressive HIV-1 infection, we assessed the correlation of DPP4+CD4+ T cells with markers of disease progression. A strong correlation for CD4 counts and a trend towards an inverse correlation with viral load were observed in female, but not in male PLHIV. No association could be identified for CD8+ CD38+ T cells. Intriguingly, DPP4+ and CD38+ T cells within the CD4+ subset correlated positively in women, but not in men. Taken together, these results suggest that high CD4+DPP4+ T cell frequencies correlate with more favorable prognostic surrogate markers of HIV-1 infection in women but not in men. Recent findings showed that lower levels of soluble DPP4 were associated with a poorer prognosis in HIV-1 infection [38]. Since DPP4 is involved in T cell function and homeostasis, lower levels of CD4+DPP4+ T cells might reflect the progressive immune deterioration in HIV infection. Our data suggest that the loss of DPP4 is associated to and might be involved in this process and that its dynamics differ comparing women and men. In addition, our results suggest, that an evaluation of DPP4 as prognostic marker needs to take its sex bias into account.

An important limitation of the present study is its genetic diversity. We assessed sex differences in DPP4 on the mRNA and protein levels in a cohort of 101 individuals. All except one participant were of Caucasian race. Therefore, the present work is limited to a narrow genetic background. HIV infection is, however, a disease afflicting more non-Caucasian individuals. Future studies should include a genetically more diverse study population.

In summary, we identified sex differences in the pathobiology of T cells in HIV-1 infection using a data-driven approach. Our observations illustrate that DPP4 is a component of T cell biology involved in sex differences in chronic HIV-1 infection. This opens a new line of research on sex differences in HIV-1 pathogenesis and might have important implications for the use of DPP4 as a prognostic parameter.

## Supporting information

S1 Fig Counting algorithm for gene occurrences in abstracts obtained from PubMed. The algorithm uses a list of genes (here sex biased genes as identified by analysis of microarray data) and counts the occurrences of these genes (their names, symbols and respective synonyms) in lists of abstracts. The abstracts were obtained from PubMed. The result is provided as a table of gene occurrences per list of abstracts.
(TIF)

S2 Fig Gating hierarchy for DPP4+ T cells. Exemplary gates (upper panel) with gating hierarchy (lower panel) for the identification of DPP4+ B and T cells by flowcytometry.
(TIF)

S3 Fig Expression of sex biased genes in three microarrays. Expression of 31 consistently and significantly sex biased genes is shown. Each bar represents one microarray experiment. A positive log fold change (logFC) indicates higher expression in women.
(TIF)

S4 Fig Difference of DPP4 expression comparing HI and PLWH. DPP4 surface expression was measured on CD4+ and CD8+ T cells by flowcytometry in PBMC. PBMC were obtained from 19 healthy controls (10 women, 9 men), 24 controllers (14 women, 10 men) and 44 progressors (19 women, 25 men). Frequencies of CD4+DPP4+ and CD8+DPP4+ T cells were compared between groups. P values were calculated using a two-sided Wilcoxon signed rank test.
(TIF)

S5 Fig Correlation matrix. Surface expression of DPP4 on CD4 and CD8 T cells measured by flowcytometry, viral load, CD4 T cell counts and CD38 expression on CD4 and CD8 T cells were correlated with each other. Samples were obtained from 68 PLWH (33 women, 35 men). Spearman‘s rho was computed and tested for statistical significance. Blue ellipses represent positive, whereas red ellipses indicate negative correlations of the respective variables. The Spearman’s rho is displayed in the center of each ellipse. P values were adjusted for multiple comparisons. Non-significant correlations after adjustment are crossed out.
(TIF)

S6 Fig Correlation of DPP4+CD4+ T cells with CD4+ T cell activation. DPP4+CD4+ T cells were correlated with CD4 T cell activation as measured by the surface expression of CD38. Samples were obtained from 68 persons living with HIV-1 (33 women, 35 men). Loess regression lines with 95% confidence intervals were added. The correlation coefficient r and the p-value were computed using the Spearman method.
(TIF)

S1 Table Gene ontology categories. The table shows GO categories with number of overlapping genes and respective *p-*values. *P-*values were adjusted for multiple comparisons using the Benjamin-Hochberg method and were included as *q*-values. Only significant overlaps (q ≤ 0.05) are shown.
(TIF)

S2 Table PubMed abstracts with PubMed search details. The table shows the search details for each PubMed search and the number of obtained abstracts.
(TIF)

S3 Table RT-qPCR primers. The table shows the primers which were used for RT-qPCR. The primer sequences for HMBS and HPRT1 were obtained from Vandesompele et al. [8].
(TIF)

S4 Table Microarray data and literature mining. The table shows the integrated results of microarray data and literature mining. P-values (p) and logarithmic fold changes (logFC) are given as means comparing the three microarray experiments. Genes are sorted by mean p value in ascending order. The occurrences in the searched abstracts are given for each gene.
(TIF)

S1 File Counting gene occurrences. R code for counting occurrences of gene names, symbols or synonyms in abstracts.
(HTML)
