# Supplementary figures and images for: Integration of microarray data and literature mining identifies a sex bias in DPP4+CD4+ T cells in HIV-1 infection
Source: PLoS One. 2020 Sep 18;15(9):e0239399. doi: 10.1371/journal.pone.0239399 (PMC7500694; doi:10.1371/journal.pone.0239399)

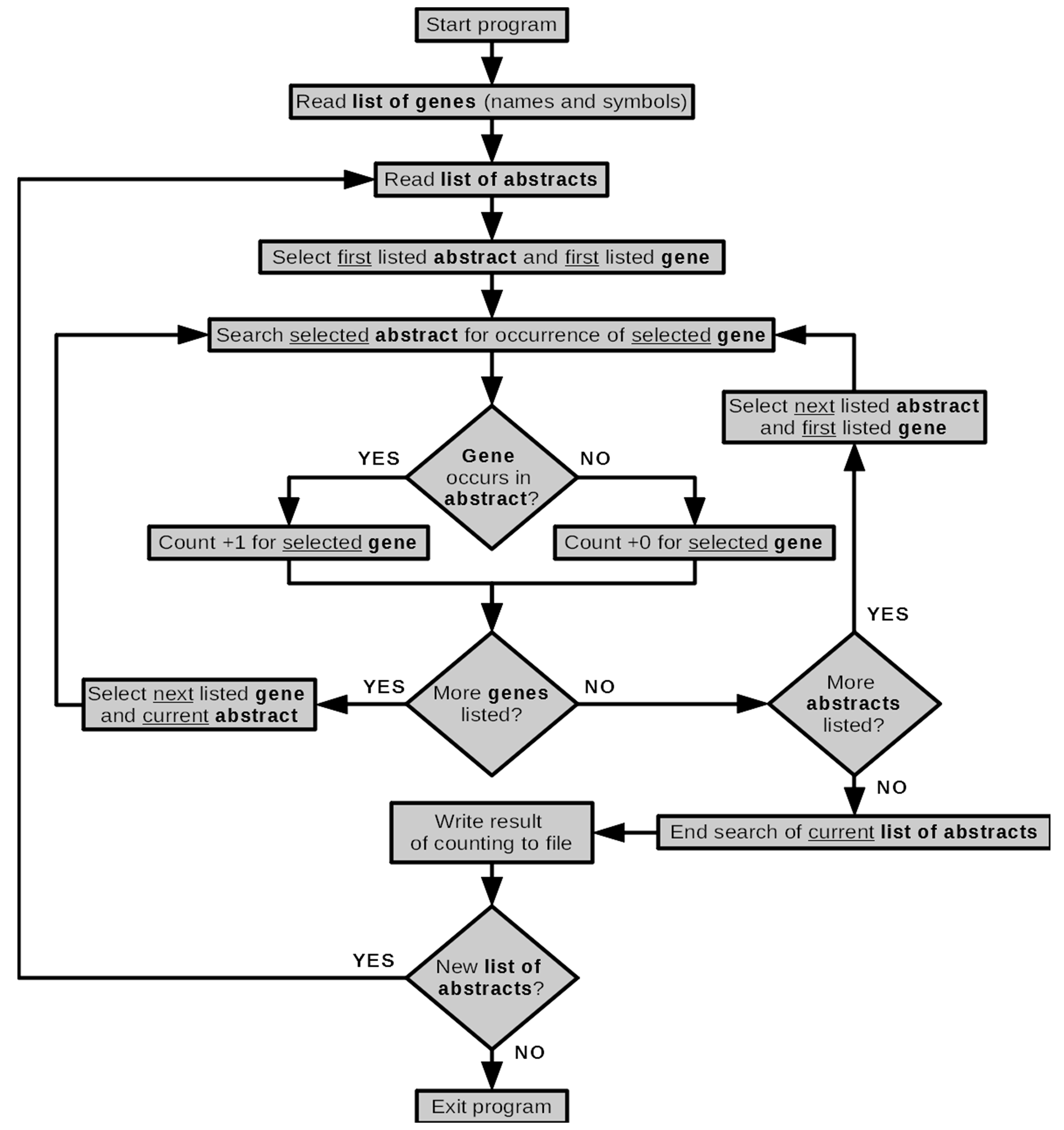

Supplement: S1 Fig — The algorithm uses a list of genes (here sex biased genes as identified by analysis of microarray data) and counts the occurrences of these genes (their names, symbols and respective synonyms) in lists of abstracts. The abstracts were obtained from PubMed. The result is provided as a table of gene occurrences per list of abstracts. (TIF) [file pone.0239399.s001.tif]

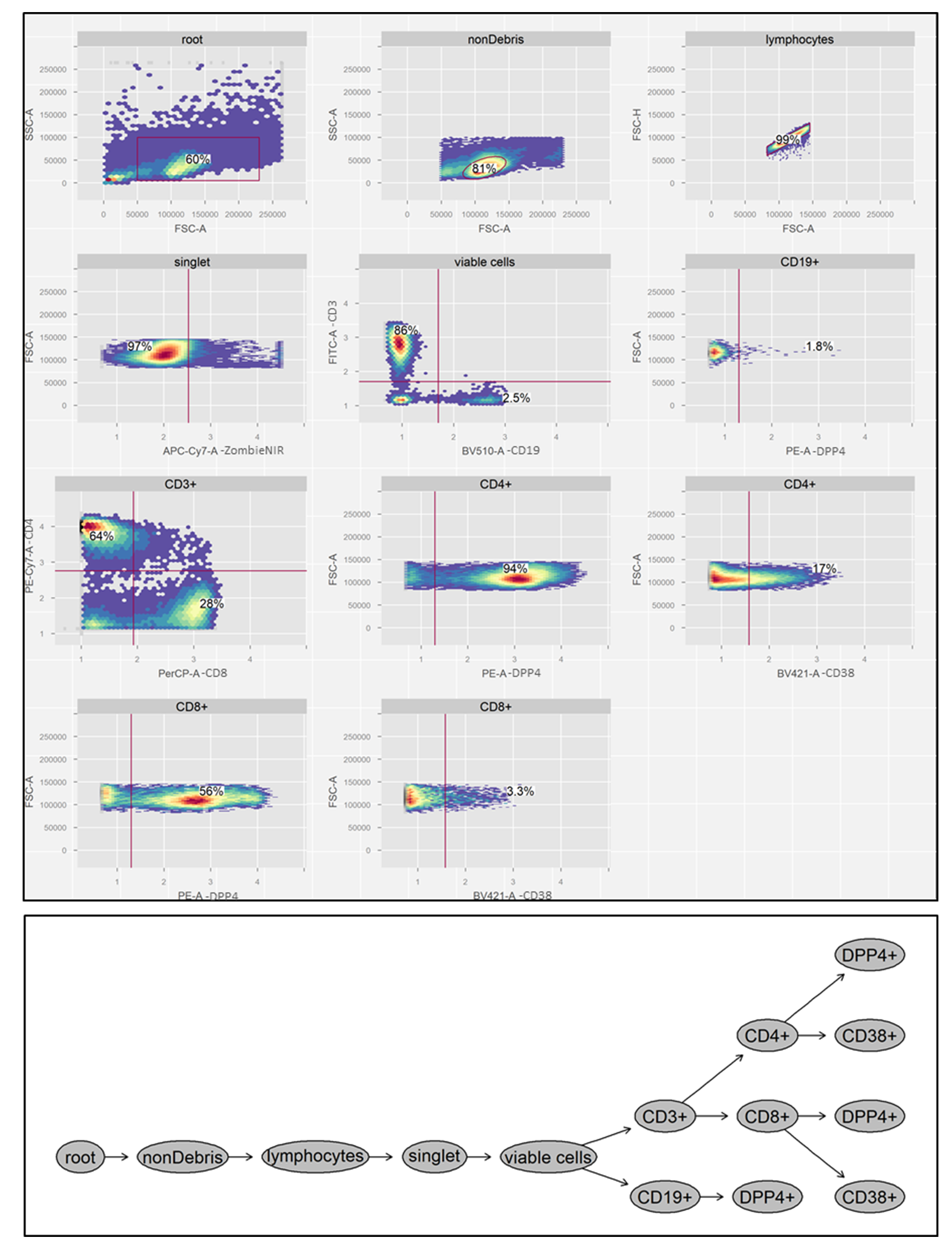

Supplement: S2 Fig — Exemplary gates (upper panel) with gating hierarchy (lower panel) for the identification of DPP4+ B and T cells by flowcytometry. (TIF) [file pone.0239399.s002.tif]

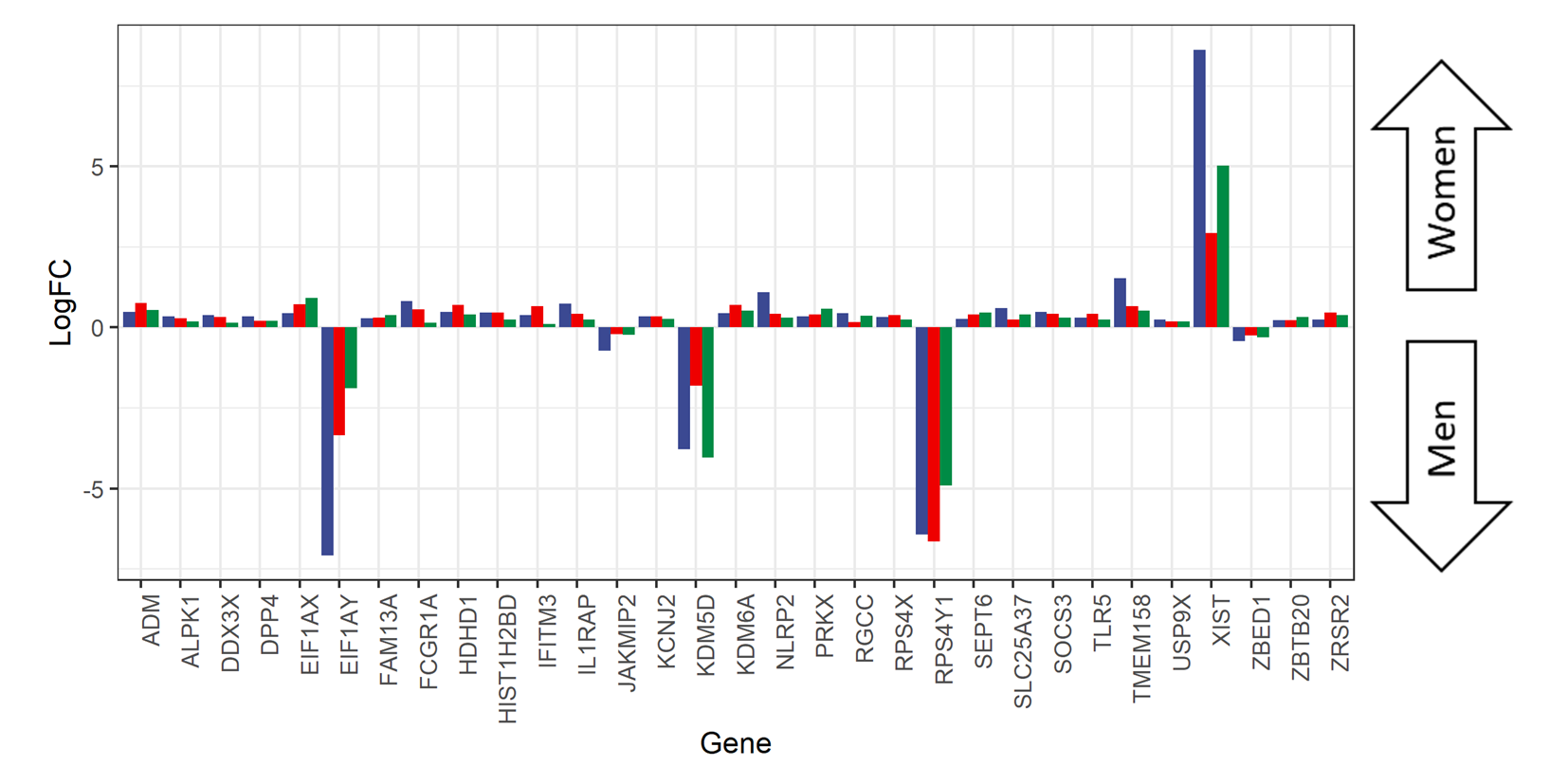

Supplement: S3 Fig — Expression of 31 consistently and significantly sex biased genes is shown. Each bar represents one microarray experiment. A positive log fold change (logFC) indicates higher expression in women. (TIF) [file pone.0239399.s003.tif]

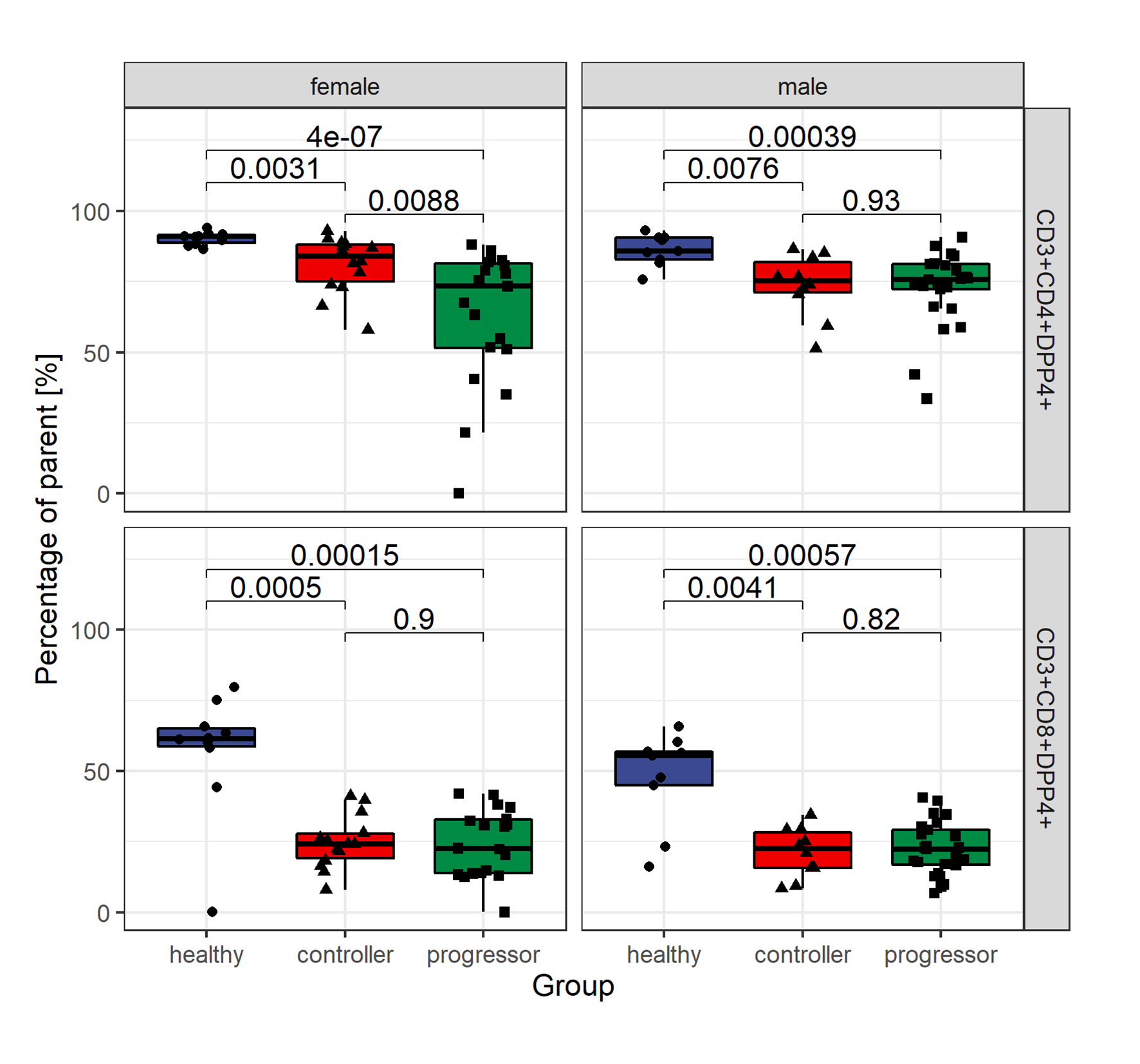

Supplement: S4 Fig — DPP4 surface expression was measured on CD4+ and CD8+ T cells by flowcytometry in PBMC. PBMC were obtained from 19 healthy controls (10 women, 9 men), 24 controllers (14 women, 10 men) and 44 progressors (19 women, 25 men). Frequencies of CD4+DPP4+ and CD8+DPP4+ T cells were compared between groups. P values were calculated using a two-sided Wilcoxon signed rank test. (TIF) [file pone.0239399.s004.tif]

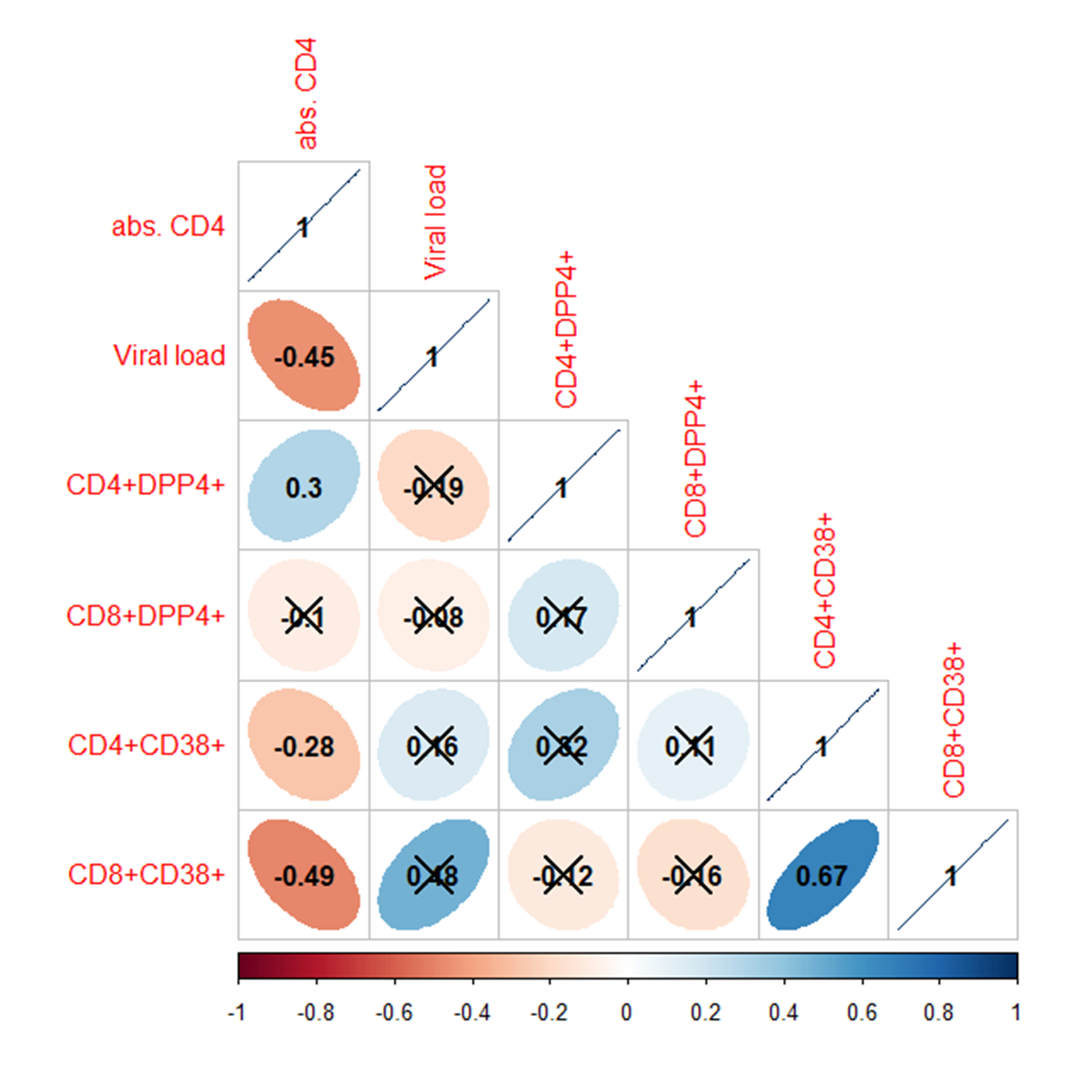

Supplement: S5 Fig — Surface expression of DPP4 on CD4 and CD8 T cells measured by flowcytometry, viral load, CD4 T cell counts and CD38 expression on CD4 and CD8 T cells were correlated with each other. Samples were obtained from 68 PLWH (33 women, 35 men). Spearman‘s rho was computed and tested for statistical significance. Blue ellipses represent positive, whereas red ellipses indicate negative correlations of the respective variables. The Spearman’s rho is displayed in the center of each ellipse. P values were adjusted for multiple comparisons. Non-significant correlations after adjustment are crossed out. (TIF) [file pone.0239399.s005.tif]

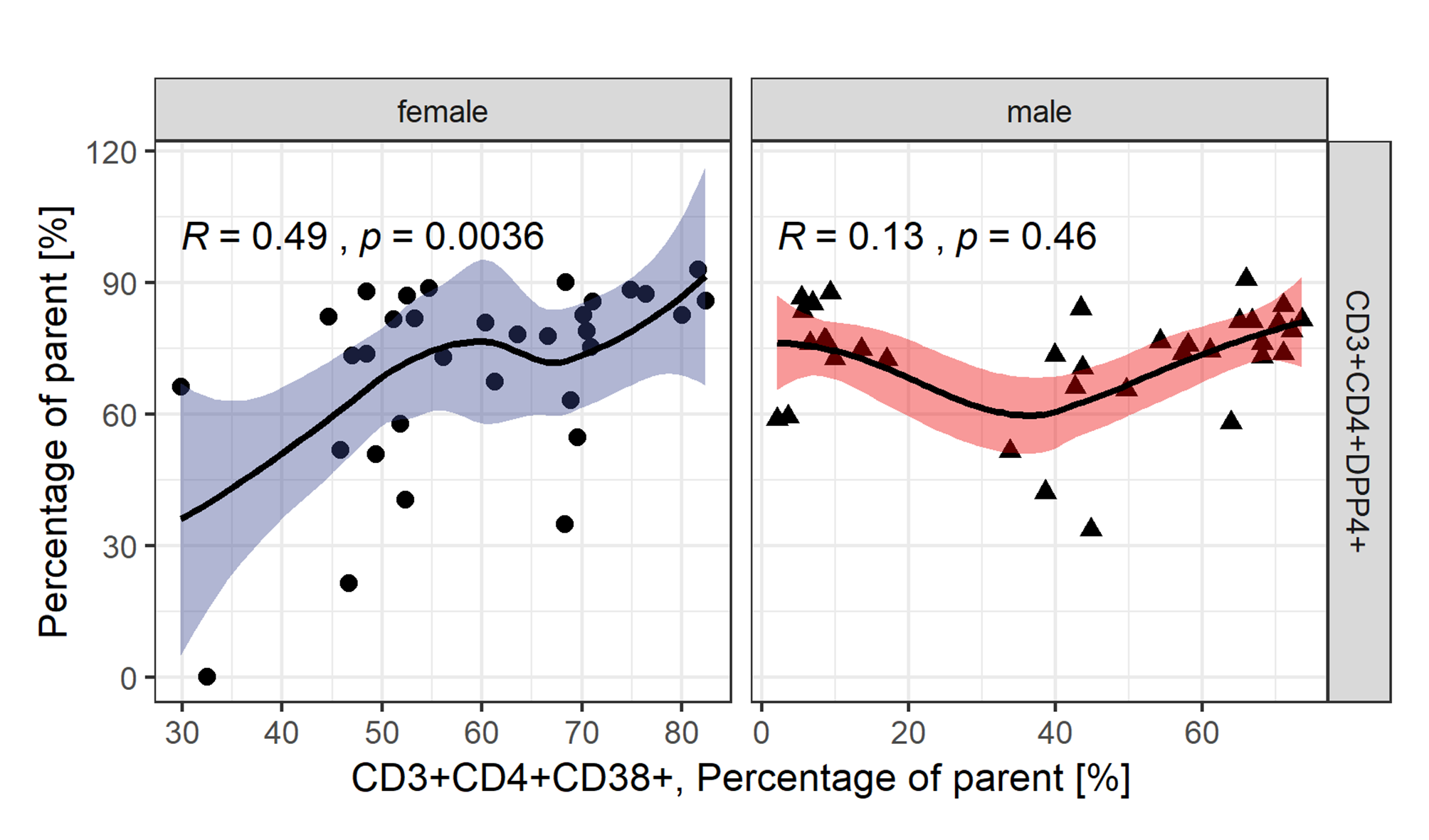

Supplement: S6 Fig — DPP4+CD4+ T cells were correlated with CD4 T cell activation as measured by the surface expression of CD38. Samples were obtained from 68 persons living with HIV-1 (33 women, 35 men). Loess regression lines with 95% confidence intervals were added. The correlation coefficient r and the p-value were computed using the Spearman method. (TIF) [file pone.0239399.s006.tif]

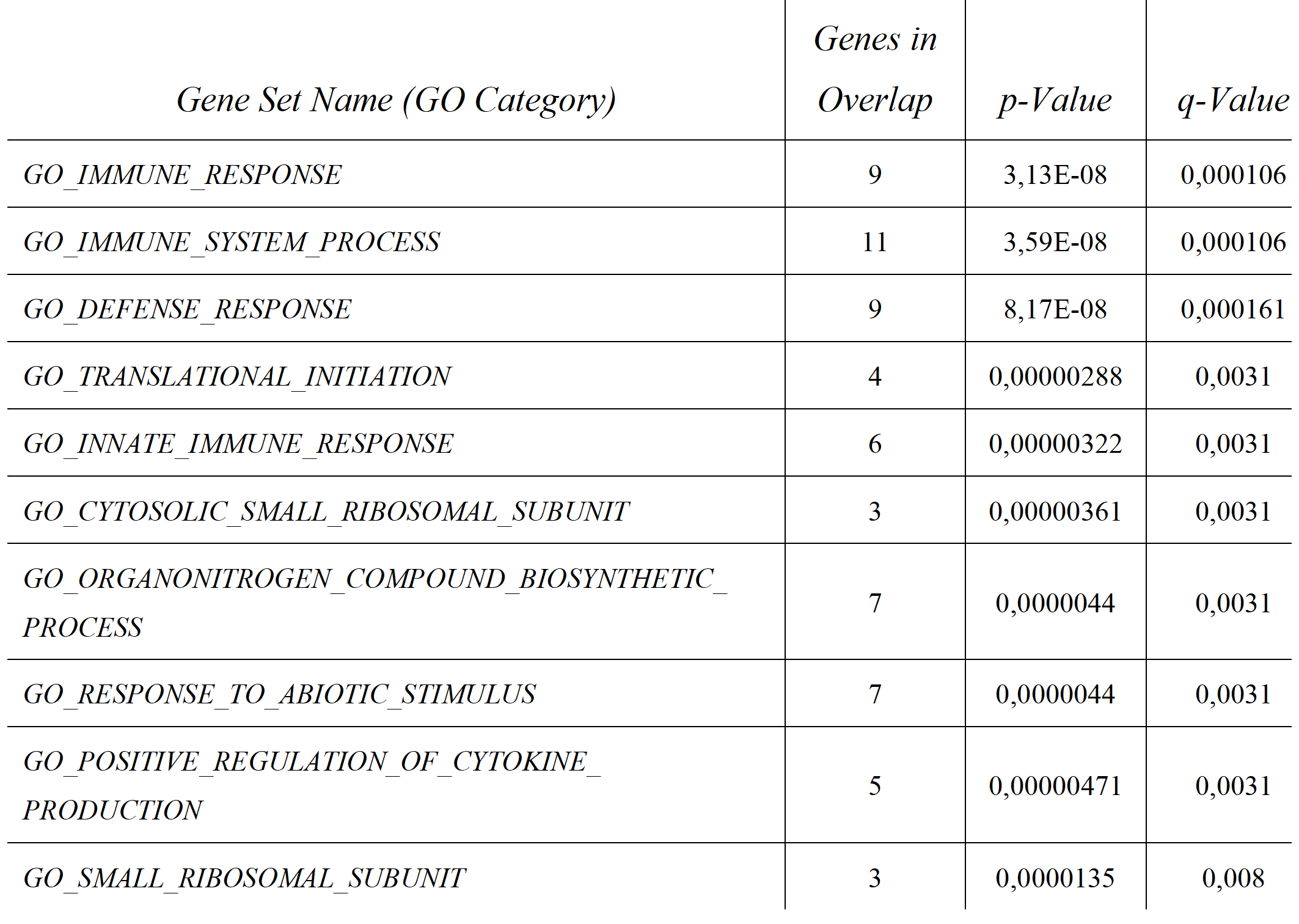

Supplement: S1 Table — The table shows GO categories with number of overlapping genes and respective p-values. P-values were adjusted for multiple comparisons using the Benjamin-Hochberg method and were included as q-values. Only significant overlaps (q ≤ 0.05) are shown. (TIF) [file pone.0239399.s007.tif]

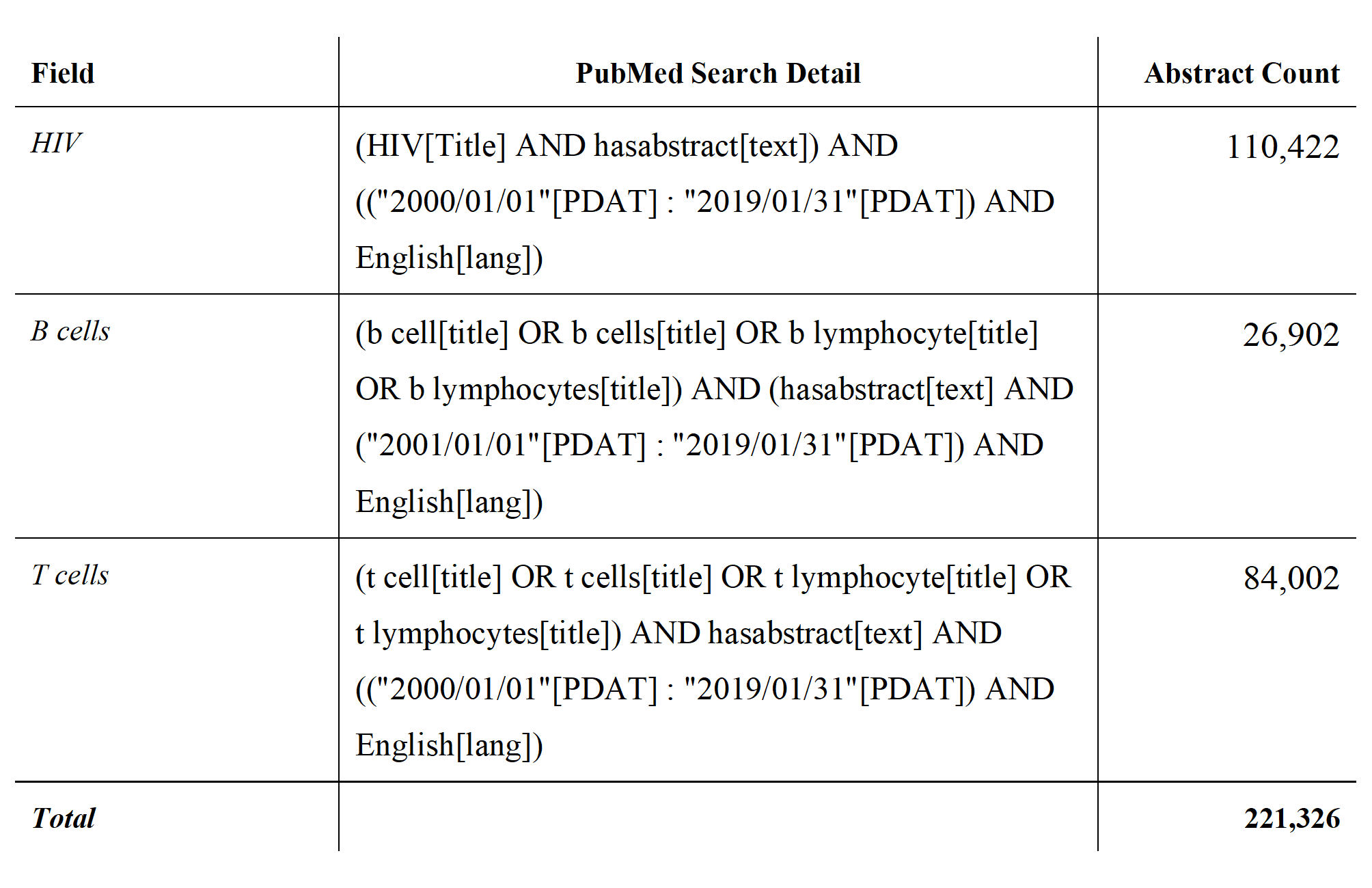

Supplement: S2 Table — The table shows the search details for each PubMed search and the number of obtained abstracts. (TIF) [file pone.0239399.s008.tif]

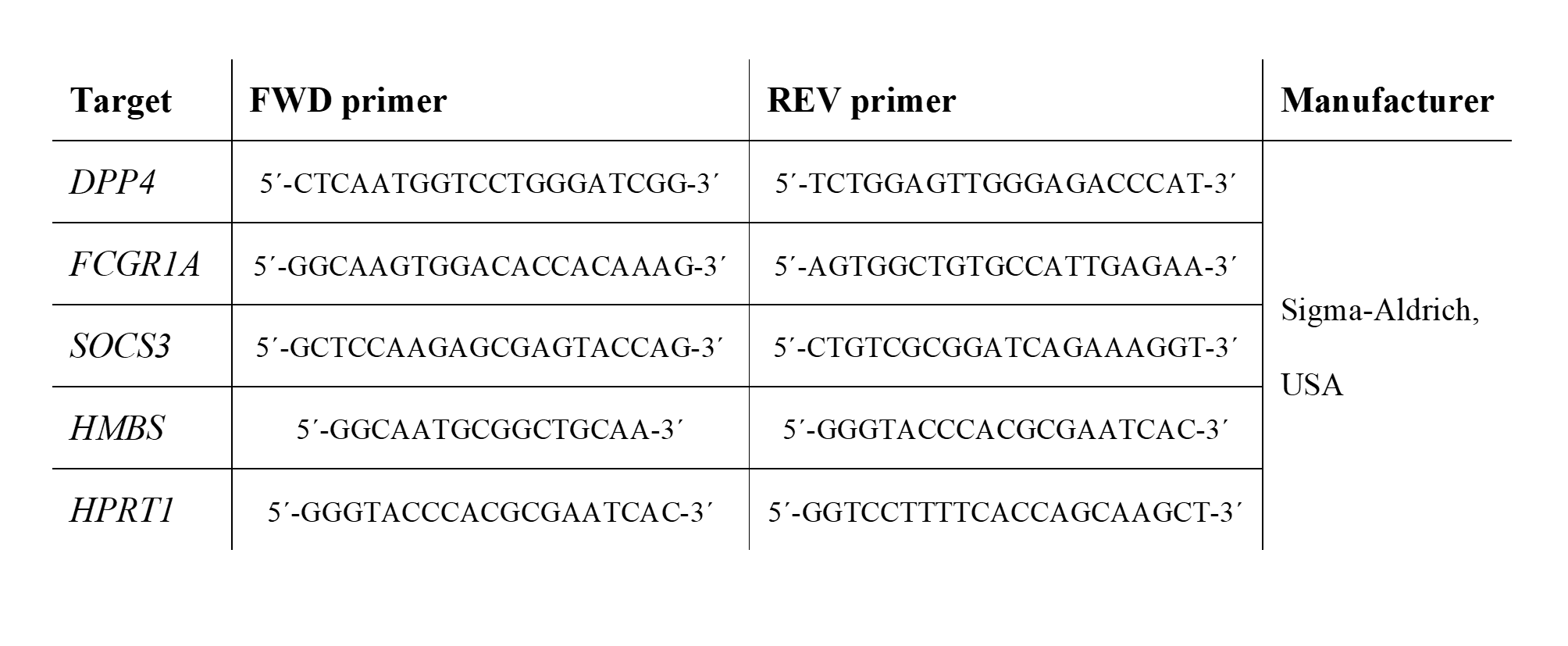

Supplement: S3 Table — The table shows the primers which were used for RT-qPCR. The primer sequences for HMBS and HPRT1 were obtained from Vandesompele et al. [8]. (TIF) [file pone.0239399.s009.tif]

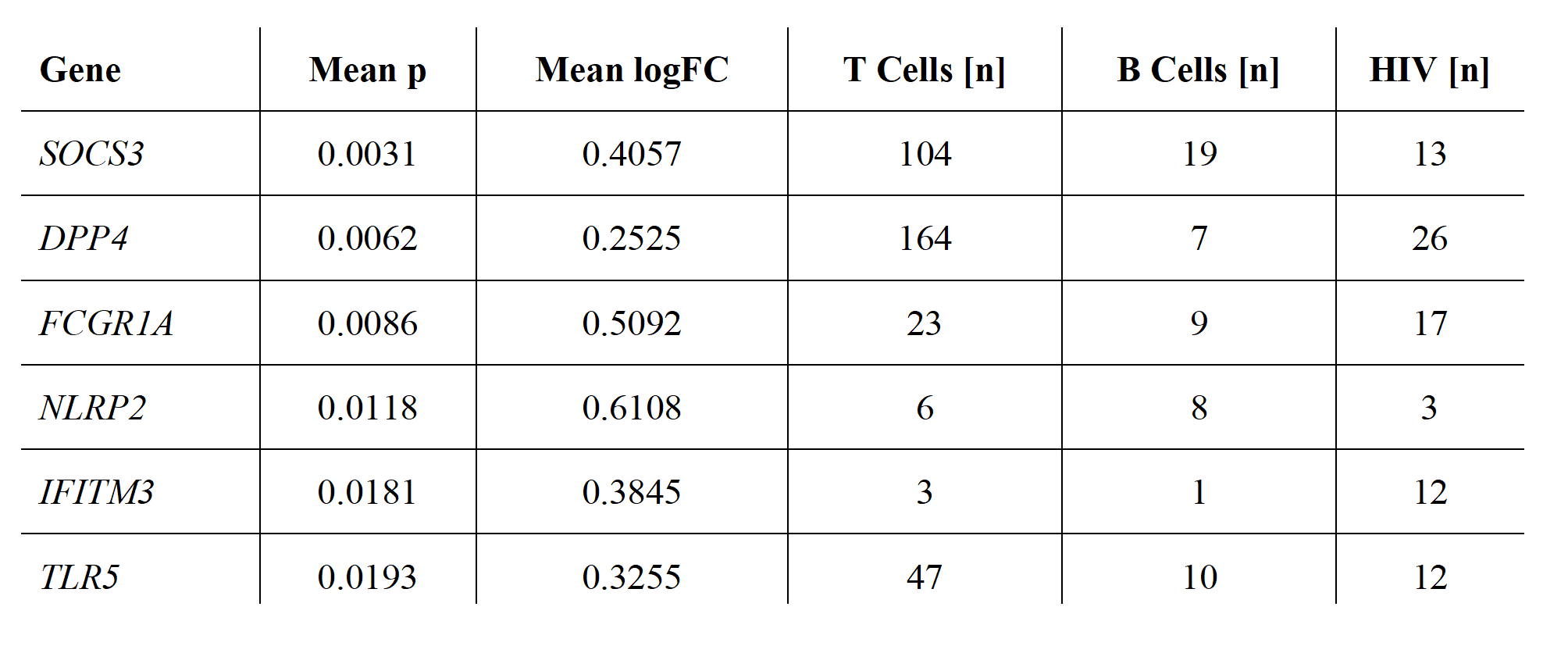

Supplement: S4 Table — The table shows the integrated results of microarray data and literature mining. P-values (p) and logarithmic fold changes (logFC) are given as means comparing the three microarray experiments. Genes are sorted by mean p value in ascending order. The occurrences in the searched abstracts are given for each gene. (TIF) [file pone.0239399.s010.tif]
