# Supplementary material for: Integration of microarray data and literature mining identifies a sex bias in DPP4+CD4+ T cells in HIV-1 infection
Source: PLoS One. 2020 Sep 18;15(9):e0239399. doi: 10.1371/journal.pone.0239399 (PMC7500694; doi:10.1371/journal.pone.0239399)
Supplement: S1 File — R code for counting occurrences of gene names, symbols or synonyms in abstracts. (HTML) [file pone.0239399.s011.html]

Counting Gene Occurrences


# Counting Gene Occurrences

### Example code

```
# Source functions
source('scripts/genefindr.R')

# Select abstract files
abstractFileNames = c("set1.txt",
                      "set2.txt",
                      "set3.txt")

names(abstractFileNames) = abstractFileNames

# Run genefindr for counting occurrences
runGeneFindr(abstractFileNames, 
             n.cores = 1, # to run on Widonws; on UNIX load package parallel
                          # and use detectCores()
             chunks = 10, # number of chunks needs to be smaller or equal 
                          # to number of investigated genes
             GEOsearchFile = "resultFromMicroarrays.csv" # investigate only 
                                                         # sex biased genes
             )
```

### Function runGeneFindr()

Reads a list with gene names, symbols and aliases, then searches the gene occurences in each abstract file and writes a file with the result for each set of abstracts. Returns a list with results as list object if required.

```
runGeneFindr = function(abstractFileNames, # vector of filenames for abstracts
                        returnResult = FALSE, # determines, if an object as 
                                              # result should be returned
                        GEOsearchFile = NULL, # Specifies if results from GEO should 
                                              # be used to subset 
                                              # protein coding genes
                        ... # additional arguments for genefindr()
                        ){
  HGNC = readGeneList(GEOsearchFile = GEOsearchFile)
  resultList = list()
  for(i in abstractFileNames){
    res = genefindr(file.name = i, geneList = HGNC$geneList, HGNCmtx = HGNC$HGNCmtx, ...)
    resultList = c(resultList, res)
  }
  names(resultList) = abstractFileNames
  if(returnResult==TRUE){
    return(resultList)
  }
}
```

### Function genefindr()

The function genefindr() searches pubmed abstracts for elements of a list of words, e.g. genes. It outputs a matrix with one column. Values are counts of occurences per abstract of each element of this list.

```
genefindr <- function(file.name, # name of abstracts file to be read
                      geneList, # a list of gene names, symbols and aliases,
                                # where each element is for one gene
                      path = "files", # path, where abstracts are stored
                      chunks = 100, # chunks in case memory is an issue
                      n.cores = detectCores(), # number of cores 
                                               # (unix only: i.e. detectCores(); 
                                               # for Windows use n.cores=1
                      lengthEntry = 3,
                      HGNCmtx = NULL
                      ){  
  
  # load required package
  if(!require(parallel)){stop("Package parallel could not be loaded!")}
  
  # Print action
  print(paste("Searching abstract file", file.name))

  # remove entries lengt <= lengthEntry OR 
  # lengt <= (lengthEntry-1) AND no number in symbol 
  if(!is.null(lengthEntry)){
    listShortRemove=function(x1){
      x2 = sapply(x1, strsplit, split = "")
      x2a = suppressWarnings(lapply(x2, as.numeric))
      x2b = lapply(x2a, is.na)
      x2c = sapply(x2b, function(x) all(x))
      x3 = sapply(x2, length) <= lengthEntry
      x4 = sapply(x2, length) <= (lengthEntry-1)
      x5 = (x3&x2c)|x4
      y=x1[!x5]
      y
    }
    geneList = lapply(geneList, listShortRemove)
    x = lapply(geneList, length) > 0
    geneList = geneList[x]
    HGNCmtx = HGNCmtx[x,]
  }
  
  # insert regular expressions of space and punctuation to each
  # element of geneList, so that each element is not part of a longer word
  geneList <- lapply(geneList, function(x){paste("[[:punct:], [:space:]]", x, "[[:punct:], [:space:]]", sep = "")})
  
  # split geneList into chunks to avoid memory problems
  if(length(geneList)/chunks < 1){stop("More chunks than geneList elements. Reduce number of chunks!")}
  start.geneList <- seq(from = 1, to = length(geneList), by = length(geneList)/chunks)
  start.geneList <- round(start.geneList, digit = 0)
  end.geneList <- c(start.geneList[-1]-1, length(geneList))
  
  # read abstract file
  file <- paste(path, file.name, sep = "/")
  abstracts <- readLines(file)
  empty.lines <- which(abstracts == "")
  abstracts <- abstracts[-empty.lines]
  
  # find ends of absrtacts by looking for PMIDs
  end.abstract <- vapply(X = "PMID", FUN = grepl, FUN.VALUE = logical(length(abstracts)), abstracts)
  
  # find ending and starting positions of abstracts
  abstract.count <- sum(end.abstract)
  end.position <- which(end.abstract == TRUE)-1
  start.position <- c(1, end.position[-length(end.position)]+1)+2
  
  # check consistency of starts and endings
  nothing.wrong <- end.position - start.position > 0
  ab.no <- 1:abstract.count
  ab.no <- ab.no[nothing.wrong]
 if(sum(!nothing.wrong) > 0){
   print(paste(sum(!nothing.wrong), " of ", length(nothing.wrong), " abstracts failed consistency check!"))
 }else{print("Abstract consistency check passed without problems!")}
  
  # format abstracts into one character vector per abstract
  fun1 <- function(i, abstracts, start.position, end.position){
    index.abs <- start.position[i]:end.position[i]
    abstract <- paste(abstracts[index.abs], collapse = " ")
    abstract
  }
  abstract <- vapply(X = ab.no, FUN = fun1, FUN.VALUE = character(1), abstracts, start.position, end.position)
  
 
  ## search abstracts for terms given by geneList
  # this is the bottel-neck of this function
  # geneList deals with geneList elements
  # which of length one like grepl,
  # elements with length > 1 are returned 
  # as TRUE if found in the respective abstract.
  
  # Function decides whter the letter case may be 
  # ignored: TRUE, if at least 4 characters AND 
  # at least one number
  decideIgnoreCase = function(x1){
    x2=gsub(pattern = "[[:punct:], [:space:]]", x = x1, replacement = "", fixed = TRUE)
    x3=unlist(strsplit(x2, split = ""))
    y = length(x3) > 3 & sum(!is.na(suppressWarnings(as.numeric(x3)))) > 0
    y
  }
  
  # internal function of my.grep, which desciedes, 
  # wheter to ignore cases if more pattern with length > 1
  # is passed to my.grepl
  grepl.int = function(pattern, x){
    grepl(pattern, x, useBytes = FALSE, fixed = FALSE, perl = TRUE, ignore.case = decideIgnoreCase(pattern))
  }
  
  my.grepl <- function(pattern, x){
    if(length(pattern) == 1){
      grepl(pattern, x, useBytes = FALSE, fixed = FALSE, perl = TRUE, ignore.case = decideIgnoreCase(pattern))
    }else{
      int1 <- vapply(X = pattern, FUN = grepl.int, FUN.VALUE = logical(length(x)), x)
      int1 <- apply(int1, c(1), function(x){sum(x) >= 1})
      int1
    }
  }
  
  # search abstracts for elements of geneList
  count <- c(0)
  final <- c()
  print(paste(paste(Sys.time(), "h", sep = ""), "-", "start:", count, "of", length(start.geneList)))  
  for(i in 1:length(start.geneList)){
    geneList.snip <- geneList[start.geneList[i]:end.geneList[i]]
      
    # run multicore (only unix-based systems;
    # for Windows n.core = 1!)
    print(paste("Running on", n.cores, "core(s)."))
    final.m <- mclapply(
      geneList.snip,
      my.grepl,
      abstract, 
      mc.cores = n.cores)
    gc()
    final.m <- lapply(final.m, sum)
    final <- c(final, final.m)
    count <- count+1
    print(paste(paste(Sys.time(), "h", sep = ""), "-",count, "of", length(start.geneList)))
  }
  
  # convert final to matrix, so that it 
  # can be saved by write.table. return a matrix
  dat <- data.frame(occurences = unlist(final))
  rownames(dat) <- names(final)
  geneList = lapply(geneList, paste, collapse = "///")
  geneList = unlist(geneList)
  dat = cbind(dat, HGNCmtx, geneList)
  
  write.csv(dat, paste("results/result_", sub(".txt", ".csv", file.name), sep = ""))
  dat
}
```

### Function readGeneList()

This function reads a table of entries provided by HGNC and collapses them into a list of names, symbols and aliases for each gene. Its output is a list of genes for seaching with genefindr and the HGNC file in corresponding format.

```
readGeneList = function(HGNC.file = "gene_with_protein_product.csv", # filename for input HGNC.file
                        GEOsearchFile = NULL){

  # Print action
  print(paste("Reading", HGNC.file, "as geneList"))
  
  # Read table from HGNC
  HGNCtbl <- read.delim(HGNC.file)
    
  # Select required columns
  HGNCtbl <- HGNCtbl[,c("symbol", "name", "alias_symbol", "alias_name", "prev_symbol")]
  
  if(!is.null(GEOsearchFile)){
    GEOres <- read.csv(GEOsearchFile)
    # merger table
    HGNCtbl = merge(HGNCtbl, GEOres[,c("Gene.ID", "Gene.symbol")], by.x = "symbol", by.y = "Gene.symbol")
    HGNCtbl = HGNCtbl[,-ncol(HGNCtbl)]
  }
  
  # rownames
  rownames(HGNCtbl) = HGNCtbl$symbol
    
  # convert into matrix
  HGNCmtx <- as.matrix(HGNCtbl)
    
  # collapse matrix to list
  HGNCmtx <- apply(HGNCmtx, c(1,2), gsub, pattern = "|", replacement = "///", fixed = TRUE)
  for(i in c("[", "]", "(", ")","+")){
    HGNCmtx <- apply(HGNCmtx, c(1,2), gsub, pattern = i, replacement = "", fixed = TRUE)
  }
  my.collapse <- function(x){
    x <- x[x != ""]
    paste(x, collapse = "///")
  }
  geneList <- apply(HGNCmtx, 1, my.collapse)
  names(geneList) <- rownames(HGNCmtx)
    
  # remove duplicates from geneList 
  if(sum(duplicated(geneList))>0){
    geneList <- geneList[!duplicated(geneList)]
    print("duplicates removed")
  }
  
  # split elements by "///"
  geneList <- sapply(geneList, strsplit, split = "///")
  
  # remove dulpicates within each list entry
  geneList = lapply(geneList, function(x) x[!duplicated(x)])
  list(geneList=geneList, HGNCmtx=HGNCmtx)
}
```

### Function count.abstracts()

This function counts the number of pubmed-abstracts in a file.

```
count.abstracts <- function(file.name, path = "files"){

  # read abstract file
  file <- paste(path, file.name, sep = "/")
  abstracts <- readLines(file)
  empty.lines <- which(abstracts == "")
  abstracts <- abstracts[-empty.lines]
  
  # find ends of absrtacts by looking for PMIDs
  end.abstract <- vapply(X = "PMID", FUN = grepl, FUN.VALUE = logical(length(abstracts)), abstracts)
  
  # find ending and starting positions of abstracts
  abstract.count <- sum(end.abstract)
  abstract.count
}
```

### Compiling

For higher speed all functions are compiled.

```
if(!require(compiler)){stop("Package compiler could not be loaded!")}
runGeneFindr <- cmpfun(runGeneFindr)
genefindr <- cmpfun(genefindr)
readGeneList <- cmpfun(readGeneList)
count.abstracts <- cmpfun(count.abstracts)
```
